# Supplementary material for: RK-33, a small molecule inhibitor of host RNA helicase DDX3, suppresses multiple variants of SARS-CoV-2
Source: Front Microbiol. 2022 Aug 25;13:959577. doi: 10.3389/fmicb.2022.959577 (PMC9453862; doi:10.3389/fmicb.2022.959577)
Supplement: Supplementary file 1 [file Data_Sheet_1.PDF]

Supplementary Methods

RNA-Seq

Total RNA was checked for integrity on a bioanalyser and stranded libraries were constructed from oligo dT purified mRNAs. RNA was fragmented and first-strand cDNA was generated using random N6-primed reverse transcription, followed by a second-strand cDNA synthesis with dUTP instead of dTTP. The synthesized cDNA was subjected to end-repair, 3' adenylated, and adaptors were ligated to cDNA fragments. The dUTP-marked strand was degraded by Uracil-DNA-Glycosylase (UDG) and the remaining strand was PCR amplified to generate a cDNA library. After heat denaturation of the library, the single strand DNA was cyclized by splint oligo and DNA ligase. This is followed by rolling circle replication and DNA nanoball synthesis and eventually sequencing on the DNBSEQ (DNBSEQ Technology) platform.

Quality control (QC) was then performed on the raw reads to determine whether the sequencing data is suitable for subsequent analysis. After QC, the filtered clean reads were aligned to the reference human genome (hg38) using HISAT2 and the statistics of the mapping rate and the distribution of reads on the reference sequence were used to determine whether the alignment result passes the second QC of alignment using FastQC. This was followed by gene quantification analysis and other analysis based on gene expression such as PCA, correlations, and differential gene screening using DeSEQ2. We also performed significant enrichment analysis of GO function on differentially expressed genes and significance enrichment analysis of pathways analysis using REACTOME.

Proteomics

Proteins (50 ug) were reduced with 50mM Dithiothreitol in 10 mM Triethylammonium bicarbonate (TEAB) at 60C for 45 minutes followed by alkylating with 100 mM Iodoacetamide in 10 mM TEAB at room temperature in the dark for 15 minutes. MS interfering reagents were removed by precipitating 50 ug proteins by adding 8 volumes of 10% trichloroacetic acid in cold acetone at -20C for 2 h. The pellet was centrifuged at 16,000 g for 10 minutes at 4C. The TCA/Acetone supernatant was removed, and the protein pellet was washed with an equivalent 8 volumes acetone at -20C for 10 minutes prior to centrifuging at 16,000 g for 10 minutes at 4C. The acetone supernatant was removed from the protein pellet. The four sets of 16 protein pellets (50 ug)

were resuspended and digested overnight at 37C in 100 uL 50 mM TEAB with 5 ug Trypsin/Lys-C per sample. Each sample was labeled with a unique TMTpro 16-plex reagent (Thermo Fisher, LOT # VJ313476) according to the manufacturer's instructions and quenched with 5ul of 5% hydroxylamine for 15 minutes. All 16 TMT labeled peptide samples in each of the 4 sets were combined and dried by vacuum centrifugation. The combined TMT-labeled peptides (800ug) were re-constituted in 100 µL 200mM TEAB buffer and filtered through Pierce Detergent removal columns (Fisher Scientific PN 87777) to remove excess TMT label, small molecules and lipids. Peptides in the flow through were diluted to 2 mL in 10 mM TEAB in water and loaded on a XBridge C18 Guard Column (5 µm, 2.1 x 10 mm, Waters) at 250 µL/min for 8 min prior to fractionation on a XBridge C18 Column (5 µm, 2.1 x 100 mm column (Waters) using a 0 to 90% acetonitrile in 10 mM TEAB gradient over 85 min at 250 µL/min on an Agilent 1200 series capillary HPLC with a micro-fraction collector. Eighty-four 250 ul fractions were collected and concatenated into 24 fractions and dried [65]. Peptides in each of the 24 fractions were analyzed on a nano-LC-Orbitrap-Fusion Lumos-IC in FTFT mode (Thermo Fisher Scientific) interfaced with an EasyLC1200 series by reversed-phase chromatography using a 2%–90% acetonitrile in 0.1% formic acid gradient over 90 minutes at 300 nl/min on a 75 µm x 150 mm ReproSIL-Pur-120-C18-AQ column 3 µm, 120 Å (Dr.Maisch). Eluting peptides were sprayed into the mass spectrometer through a 10 µm emitter tip (New Objective) at 2.6 kV. Survey scans of precursor ions were acquired from 350-1400 m/z at 120,000 resolution at 200 m/z. Precursor ions were individually isolated within 0.7 m/z by data dependent monitoring and 15s dynamic exclusion, and fragmented using an HCD activation collision energy 34 at 50,000 resolution. Fragmentation spectra were processed by Proteome Discoverer v2.4 (PD2.4, ThermoFisher Scientific) and searched with Mascot v.2.8.0 (Matrix Science, London, UK) against RefSeq2021_204 Human database. Search criteria included trypsin enzyme, one missed cleavage, 3 ppm precursor mass tolerance, 0.01 Da fragment mass tolerance, with TMTpro on N-terminus and carbamidomethylation on C as fixed and TMTpro on K, oxidation on M, deamidation on N or Q as variable modifications. Peptide identifications from the Mascot searches were processed within PD2.4 using Percolator at a 5% False Discovery Rate confidence threshold, based on an auto-concatenated decoy database search. Peptide spectral matches (PSMs) were filtered for Isolation Interference <30%. Relative protein abundances of identified proteins were determined in PD2.4 from the normalized median ratio of TMT reporter ions, having

signal to noise ratios >1.5, from all PSMs from the same protein. Technical variation in ratios from our mass spectrometry analysis is less than 10% [66].

Supplementary Figure Legends

Figure S1.

a. PCA plot of RK-33 treated virus infected samples (blue) and DMSO control treated virus infected samples (orange). PV means "Proportion of variance", SD means standard deviation.

b. PCA plot of RK-33 treated virus infected samples (red), DMSO control treated virus infected samples (blue), and DMSO treated uninfected samples (green). PV means "Proportion of variance", SD means standard deviation.

c. A correlation plot to study the correlation of gene expression between samples. Pearson correlation coefficients of all gene expression between every sample pair was calculated, and these coefficients were plotted in the form of a heatmap. The correlation coefficients demonstrate similarity of overall gene expression between each sample with higher correlation coefficient being more similar the gene expression level. Higher correlation coefficients are represented by darker colors while lighter colors represent lower correlations.

Figure S2

Enriched pathways obtained by GSEA of DMSO treated infected samples and DMSO treated uninfected samples. GSEA was performed using the Molecular Signatures Database collection Gene Ontology (GO) Biological Process. Displayed are the top five pathways enriched in DMSO treated samples and top five enriched in uninfected DMSO samples.

Figure S3

Enriched pathways obtained by GSEA of RK-33 treated virus infected samples and DMSO treated virus infected samples.

Figure S4

a. Venn diagram displaying gene sets dysregulated by RK-33 treatment of virus (Inf RK-33 v Inf DMSO) compared to genes dysregulated by RK-33 alone (Uninf RK-33 v Uninf DMSO).

b. A Venn diagram displaying gene set comparison between all sample of all major comparisons.

c. Venn diagrams comparing gene sets from this work with published data from Calu-3 cells.

d. Venn diagrams comparing our gene sets with published work from COVID-19 patient lungs extracted post-mortem.

e. Venn diagram of differentially expressed genes from RNA-Seq analyzed samples. The left bar graph displays the number of genes, and the Y-axis represents the name of gene set. In the upper right histogram, the X-axis displays the intersection of different gene sets, and the Y-axis shows the number of genes. Each column in the lower right shows the relationship between the left gene set and the upper intersection and the corresponding number of genes in common.

Figure S5

Scatterplot displaying the QC of the RNA-seq of all the samples mapped to the human genome. Left Y-axis displays the percent of uniquely mapping reads (green) and the right Y-axis displays total reads in millions (blue).

Figure S6

a. Volcano plot of significantly changed proteins ($P<0.05$, $FC>1.1$) in RK-33 treated uninfected samples compared to DMSO treated uninfected samples.

b. Volcano plot of significantly changed proteins ($P<0.05$, $FC >1.1$) in RK-33 treated infected samples compared to RK-33 treated uninfected samples.

Figure S7

a. Venn diagram displaying proteins that are found in DMSO treated uninfected samples (DMSO control), DMSO treated infected samples (Virus), and RK-33 treated infected samples (Treated virus).

b. PCA plot of proteomics samples - DMSO treated uninfected samples (DMSO control) (blue), DMSO treated infected samples (Virus) (red), and RK-33 treated infected samples (Treated virus) (green).

Figure S8

STRING analysis of proteins showing top six Reactome enriched (up and down) pathways of RK-33 treated virus infected samples.

Figure S9

STRING analysis of proteins showing top six Reactome enriched (up and down) pathways of virus infected samples.

Table 1

A list of defining mutations present in the Alpha, Beta, Delta, and Omicron variants of SARS-CoV-2.

Figure S1

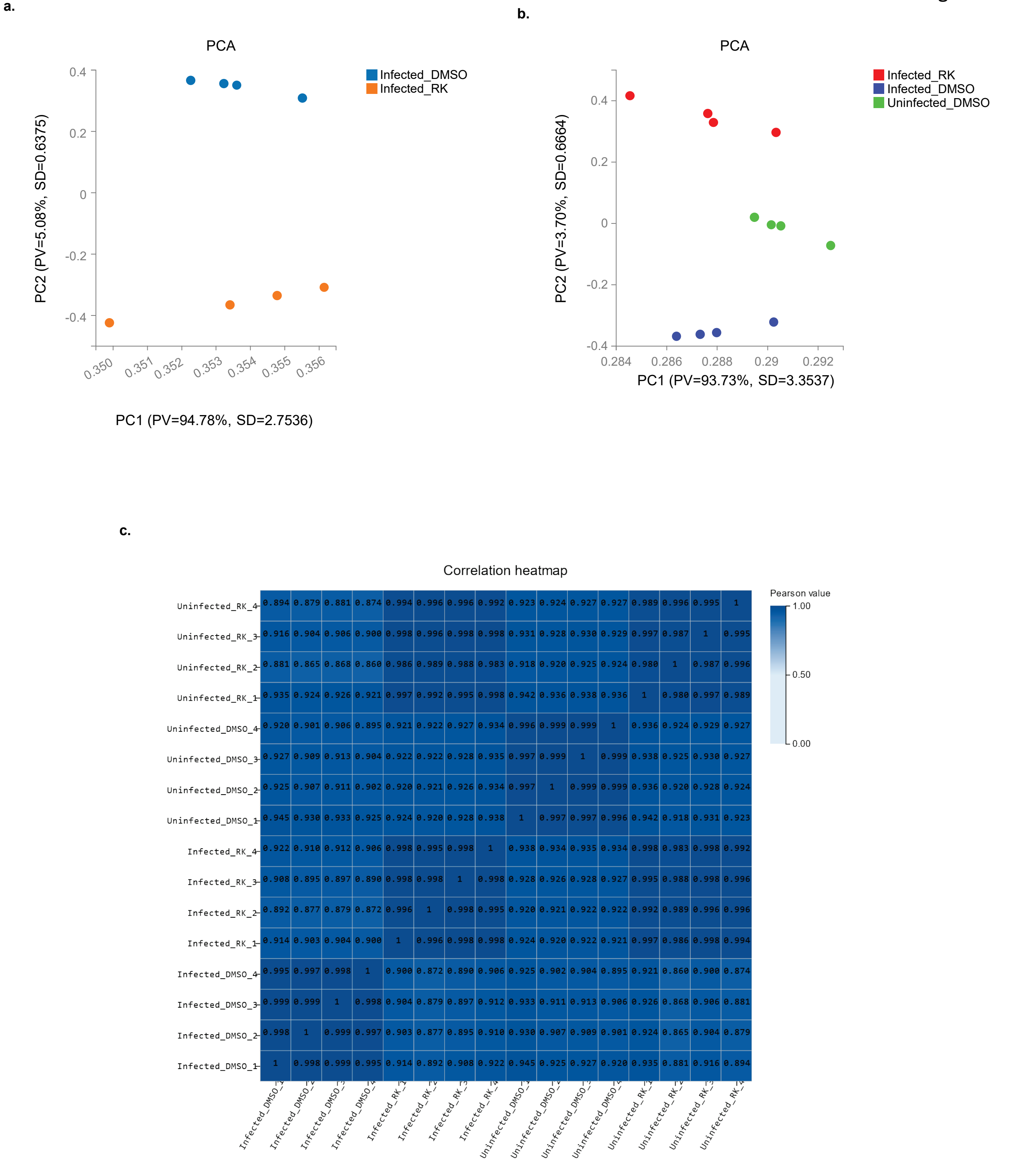
a.
PCA
Infected_DMSO
Infected_RK
PC2 (PV=5.08%, SD=0.6375)
0.4
0.2
0
-0.2
-0.4
0.350
0.351
0.352
0.353
0.354
0.355
0.356
PC1 (PV=94.78%, SD=2.7536)
b.
PCA
Infected_RK
Infected_DMSO
Uninfected_DMSO
PC2 (PV=3.70%, SD=0.6664)
0.4
0.2
0
-0.2
-0.4
0.284
0.286
0.288
0.29
0.292
PC1 (PV=93.73%, SD=3.3537)
c.
Correlation heatmap
Pearson value
1.00
0.50
0.00
Uninfected_RK_4 0.894 0.879 0.881 0.874 0.994 0.996 0.996 0.992 0.923 0.924 0.927 0.927 0.989 0.996 0.995 1
Uninfected_RK_3 0.916 0.904 0.906 0.900 0.998 0.996 0.998 0.998 0.931 0.928 0.930 0.929 0.997 0.987 1 0.995
Uninfected_RK_2 0.881 0.865 0.868 0.860 0.986 0.989 0.988 0.983 0.918 0.920 0.925 0.924 0.980 1 0.987 0.996
Uninfected_RK_1 0.935 0.924 0.926 0.921 0.997 0.992 0.995 0.998 0.942 0.936 0.938 0.936 1 0.980 0.997 0.989
Uninfected_DMSO_4 0.920 0.901 0.906 0.895 0.921 0.922 0.927 0.934 0.996 0.999 0.999 1 0.936 0.924 0.929 0.927
Uninfected_DMSO_3 0.927 0.909 0.913 0.904 0.922 0.922 0.928 0.935 0.997 0.999 1 0.999 0.938 0.925 0.930 0.927
Uninfected_DMSO_2 0.925 0.907 0.911 0.902 0.920 0.921 0.926 0.934 0.997 1 0.999 0.999 0.936 0.920 0.928 0.924
Uninfected_DMSO_1 0.945 0.930 0.933 0.925 0.924 0.920 0.928 0.938 1 0.997 0.997 0.996 0.942 0.918 0.931 0.923
Infected_RK_4 0.922 0.910 0.912 0.906 0.998 0.995 0.998 1 0.938 0.934 0.935 0.934 0.998 0.983 0.998 0.992
Infected_RK_3 0.908 0.895 0.897 0.890 0.998 0.998 1 0.998 0.928 0.926 0.928 0.927 0.995 0.988 0.998 0.996
Infected_RK_2 0.892 0.877 0.879 0.872 0.996 1 0.998 0.995 0.920 0.921 0.922 0.922 0.992 0.989 0.996 0.996
Infected_RK_1 0.914 0.903 0.904 0.900 1 0.996 0.998 0.998 0.924 0.920 0.922 0.921 0.997 0.986 0.998 0.994
Infected_DMSO_4 0.995 0.997 0.998 1 0.900 0.872 0.890 0.906 0.925 0.902 0.904 0.895 0.921 0.860 0.900 0.874
Infected_DMSO_3 0.999 0.999 1 0.998 0.904 0.879 0.897 0.912 0.933 0.911 0.913 0.906 0.926 0.868 0.906 0.881
Infected_DMSO_2 0.998 1 0.999 0.997 0.903 0.877 0.895 0.910 0.930 0.907 0.909 0.901 0.924 0.865 0.904 0.879
Infected_DMSO_1 1 0.998 0.999 0.995 0.914 0.892 0.908 0.922 0.945 0.925 0.927 0.920 0.935 0.881 0.916 0.894
Infected_DMSO_1
Infected_DMSO_2
Infected_DMSO_3
Infected_DMSO_4
Infected_RK_1
Infected_RK_2
Infected_RK_3
Infected_RK_4
Uninfected_DMSO_1
Uninfected_DMSO_2
Uninfected_DMSO_3
Uninfected_DMSO_4
Uninfected_RK_1
Uninfected_RK_2
Uninfected_RK_3
Uninfected_RK_4

Figure S2

Pathways enriched in DMSO treated virus infected samples

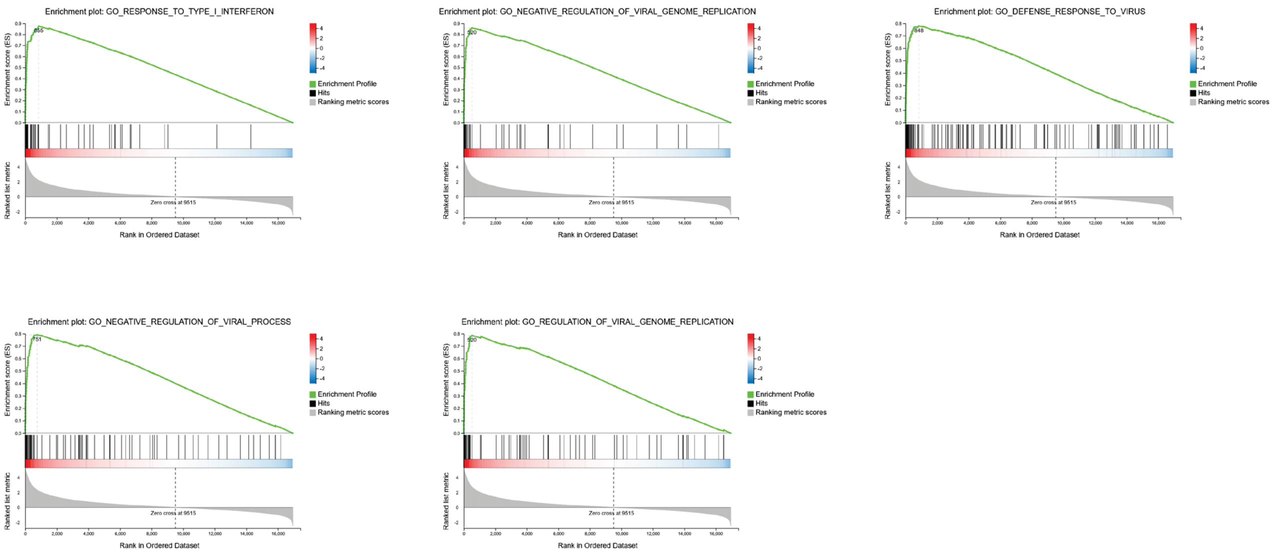

Pathways enriched in DMSO treated uninfected samples

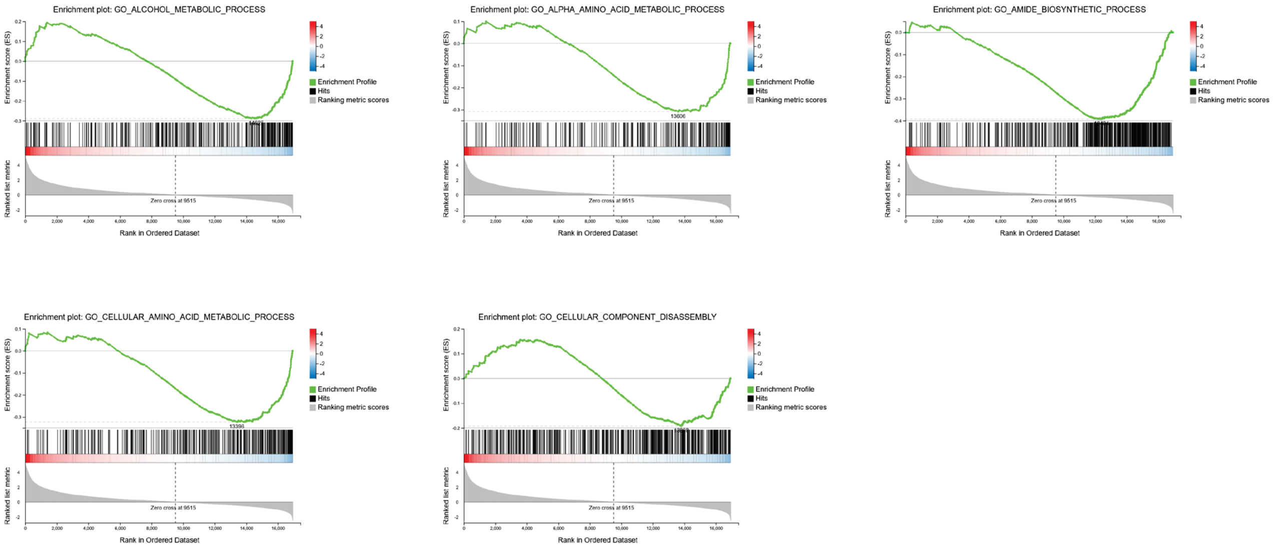

Figure S3

Pathways enriched in RK-33 treated virus infected samples

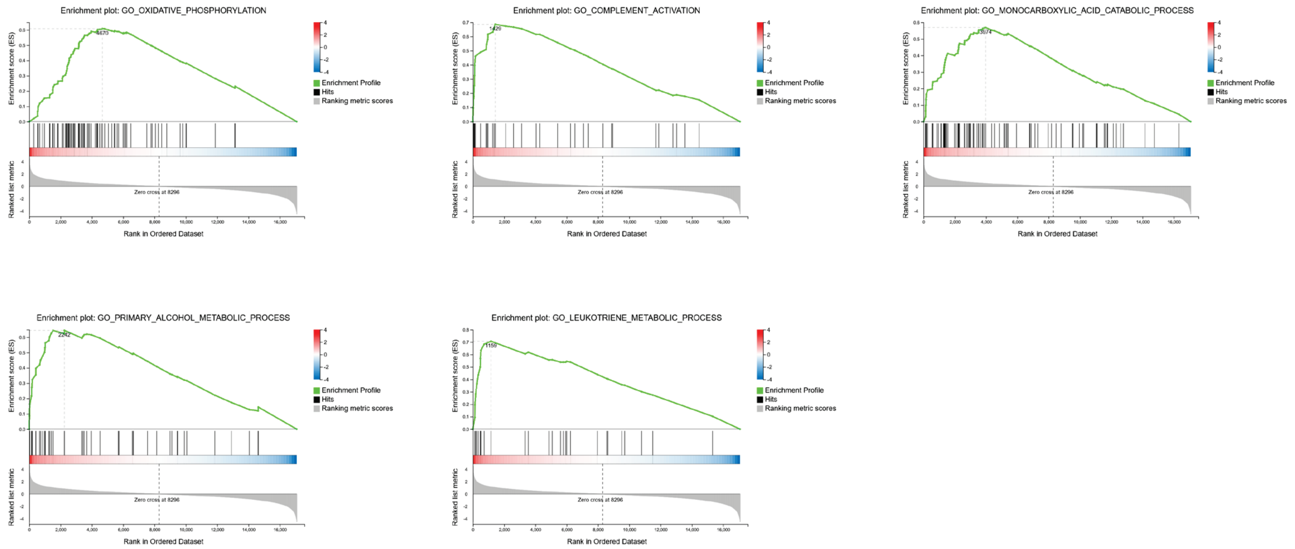

Pathways enriched in DMSO treated virus infected samples

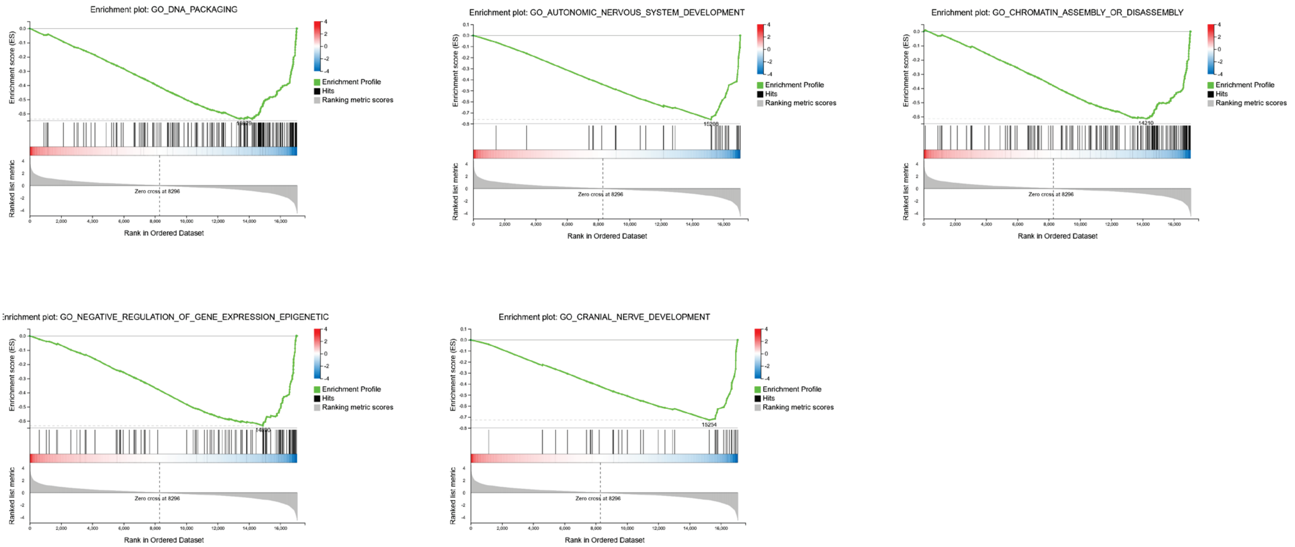

Figure S4

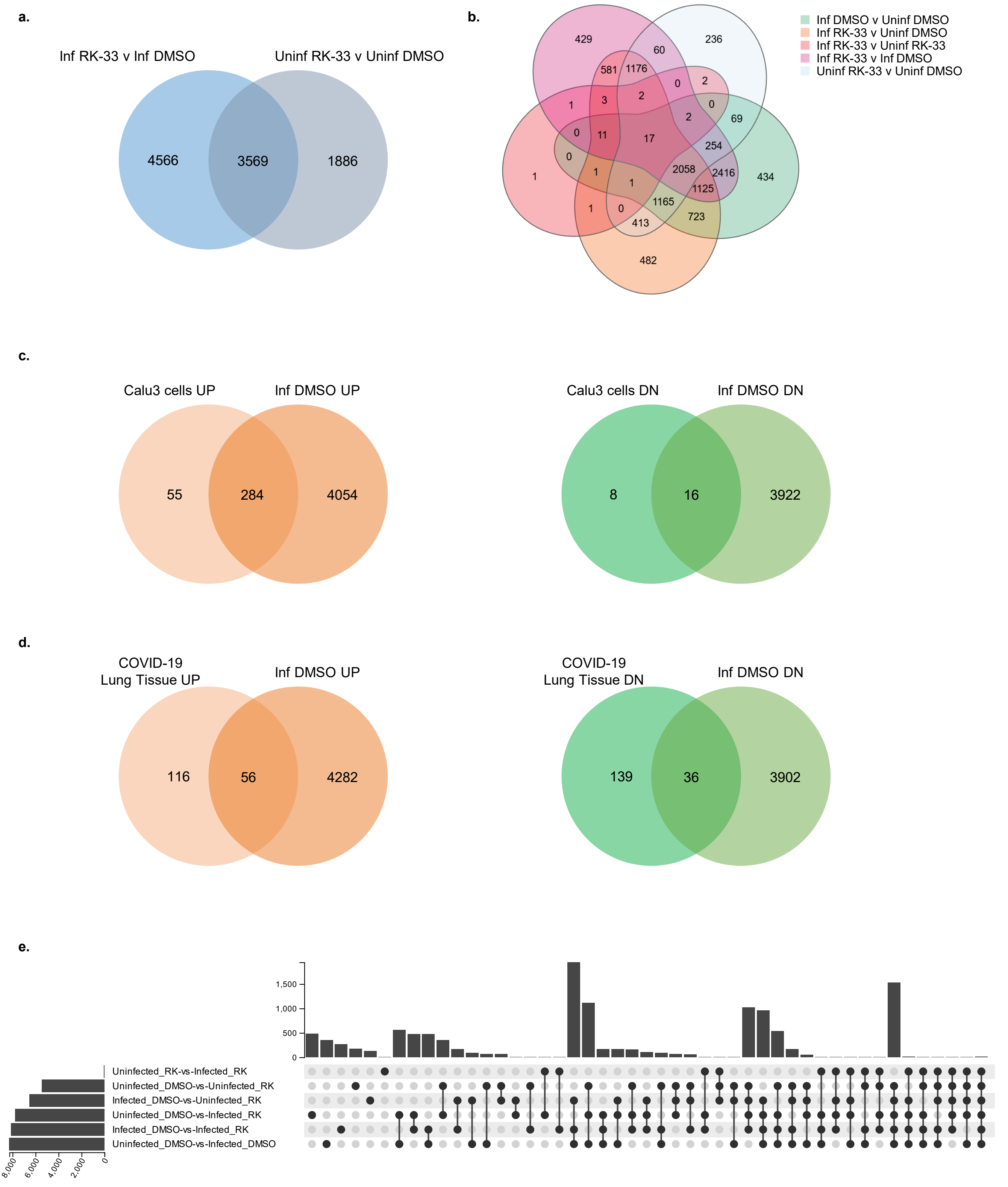

a.
Inf RK-33 v Inf DMSO
Uninf RK-33 v Uninf DMSO
4566
3569
1886
b.
Inf DMSO v Uninf DMSO
Inf RK-33 v Uninf DMSO
Inf RK-33 v Uninf RK-33
Inf RK-33 v Inf DMSO
Uninf RK-33 v Uninf DMSO
429
60
236
581
1176
0
2
1
3
2
0
69
0
11
17
2
254
0
1
1
2058
2416
1125
434
1
1165
1
0
413
723
482
c.
Calu3 cells UP
Inf DMSO UP
55
284
4054
Calu3 cells DN
Inf DMSO DN
8
16
3922
d.
COVID-19
Lung Tissue UP
Inf DMSO UP
116
56
4282
COVID-19
Lung Tissue DN
Inf DMSO DN
139
36
3902
e.
1,500
1,000
500
0
Uninfected_RK-vs-Infected_RK
Uninfected_DMSO-vs-Uninfected_RK
Infected_DMSO-vs-Uninfected_RK
Uninfected_DMSO-vs-Infected_RK
Infected_DMSO-vs-Infected_RK
Uninfected_DMSO-vs-Infected_DMSO
8,000
6,000
4,000
2,000
0

Figure S5

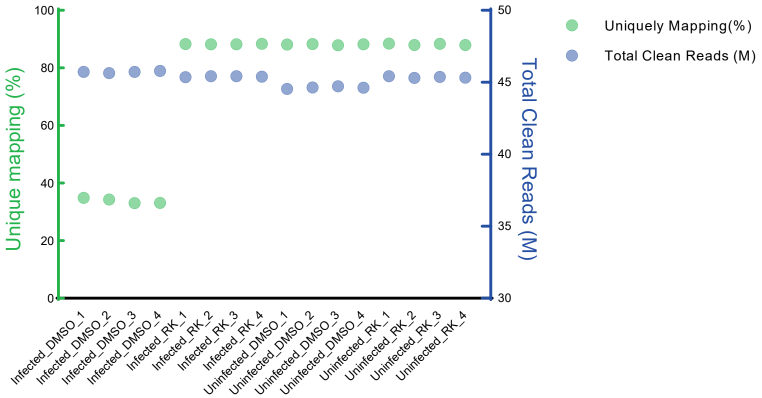

Unique mapping (%)
Total Clean Reads (M)
Uniquely Mapping(%)
Total Clean Reads (M)
100
80
60
40
20
0
50
45
40
35
30
Infected_DMSO_1
Infected_DMSO_2
Infected_DMSO_3
Infected_DMSO_4
Infected_RK_1
Infected_RK_2
Infected_RK_3
Infected_RK_4
Uninfected_DMSO_1
Uninfected_DMSO_2
Uninfected_DMSO_3
Uninfected_DMSO_4
Uninfected_RK_1
Uninfected_RK_2
Uninfected_RK_3
Uninfected_RK_4

Figure S6

a.

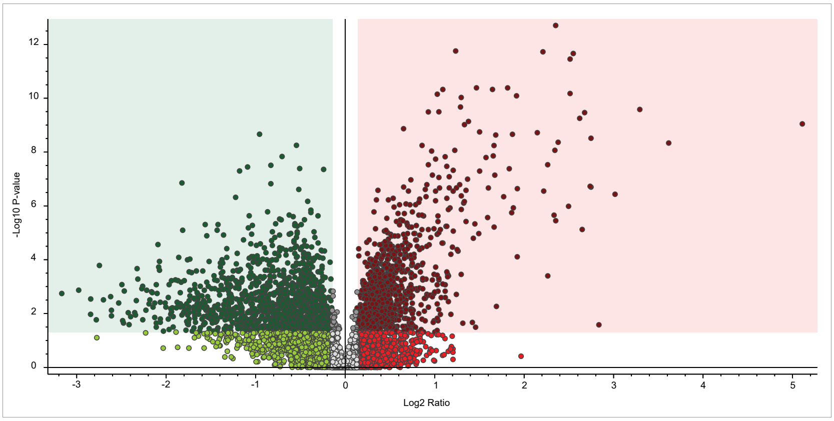

b.

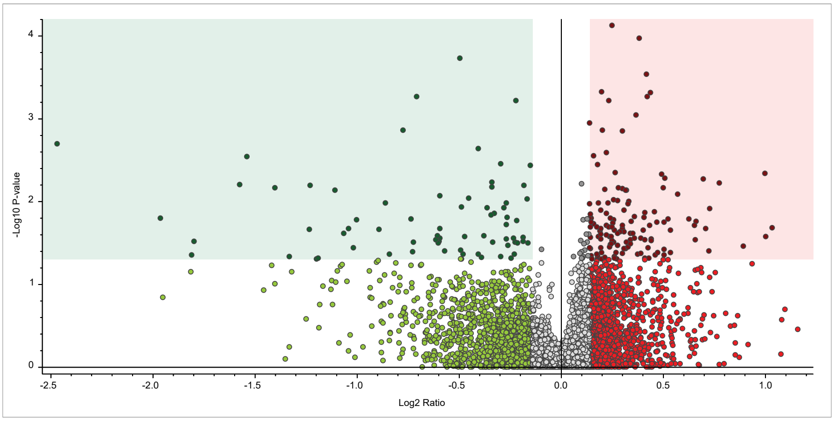

Figure S7

a.

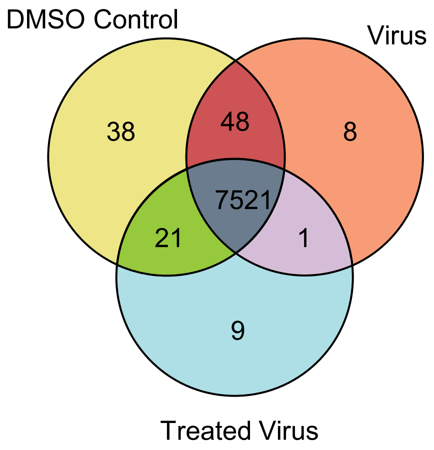

b.

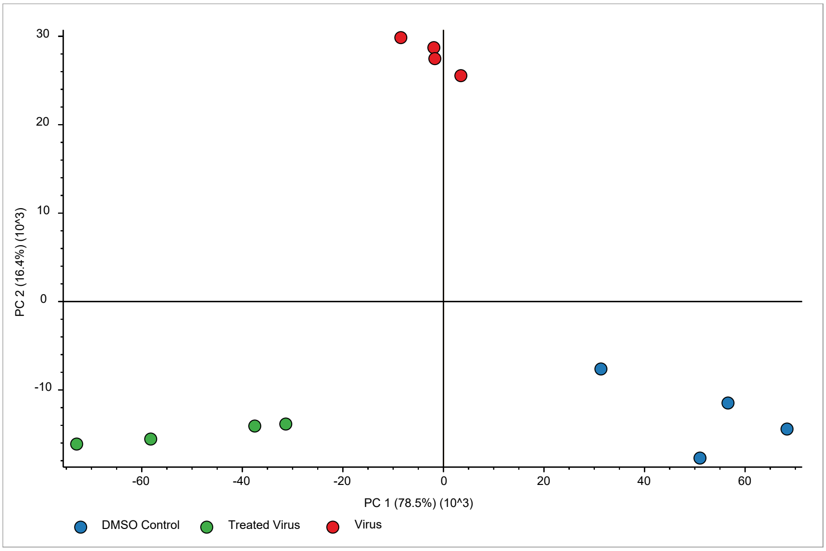

c.

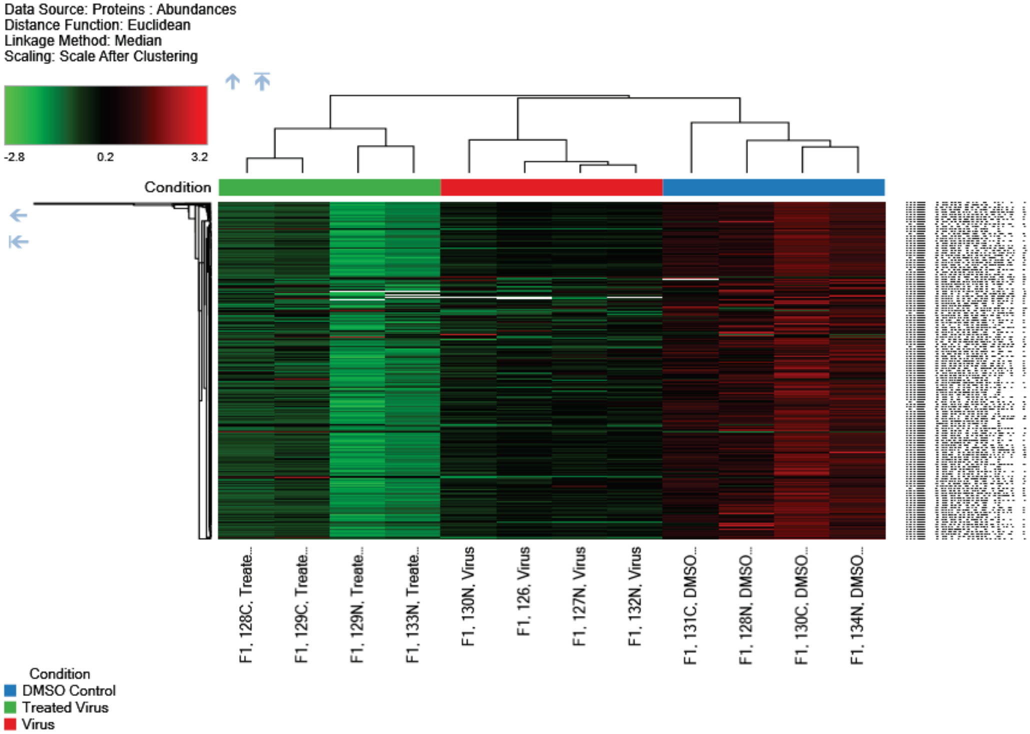

Figure S8

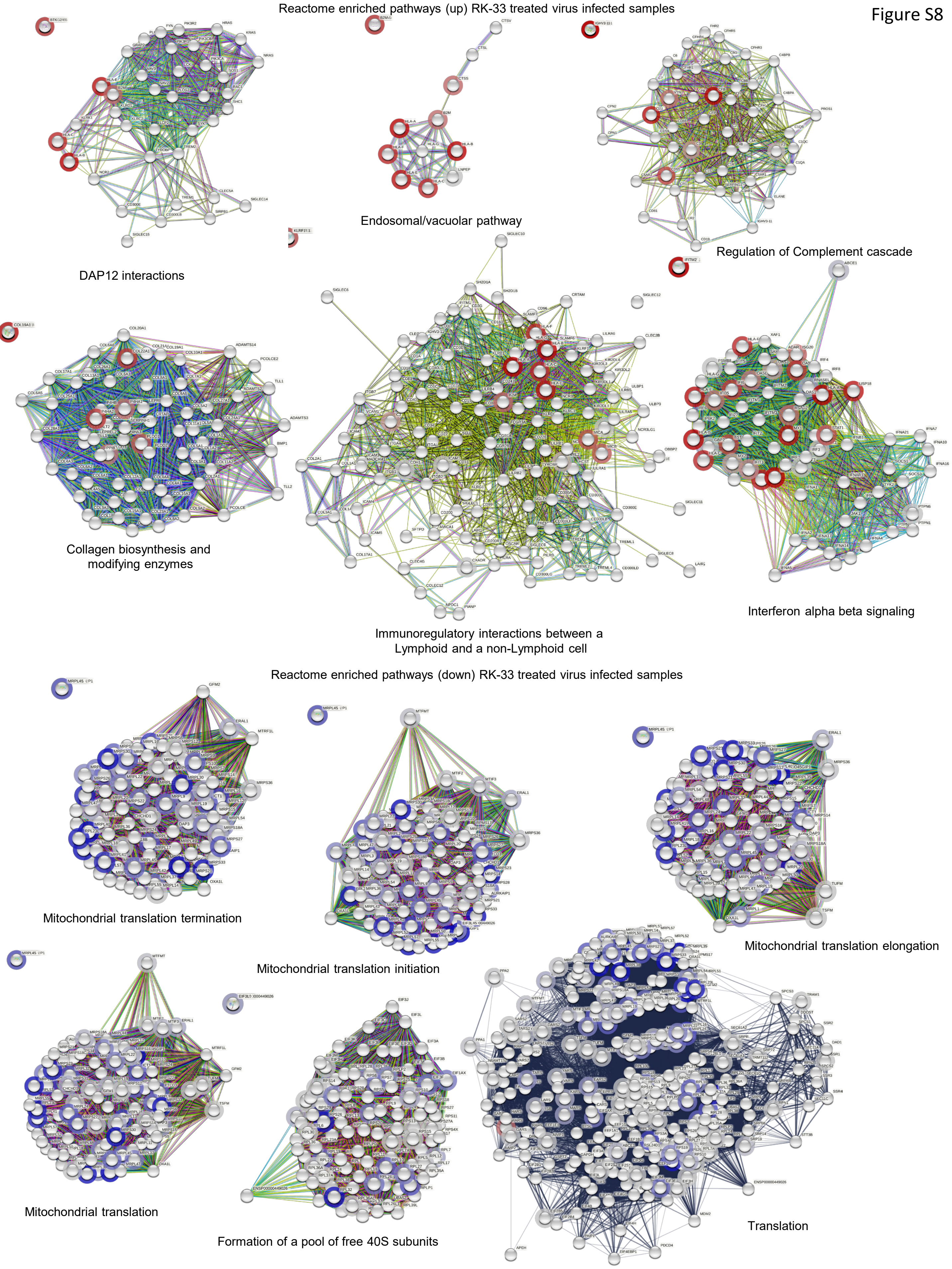

Reactome enriched pathways (up) RK-33 treated virus infected samples
DAP12 interactions
Endosomal/vacuolar pathway
Regulation of Complement cascade
Collagen biosynthesis and modifying enzymes
Immunoregulatory interactions between a Lymphoid and a non-Lymphoid cell
Interferon alpha beta signaling
Reactome enriched pathways (down) RK-33 treated virus infected samples
Mitochondrial translation termination
Mitochondrial translation initiation
Mitochondrial translation elongation
Mitochondrial translation
Formation of a pool of free 40S subunits
Translation

Figure S9

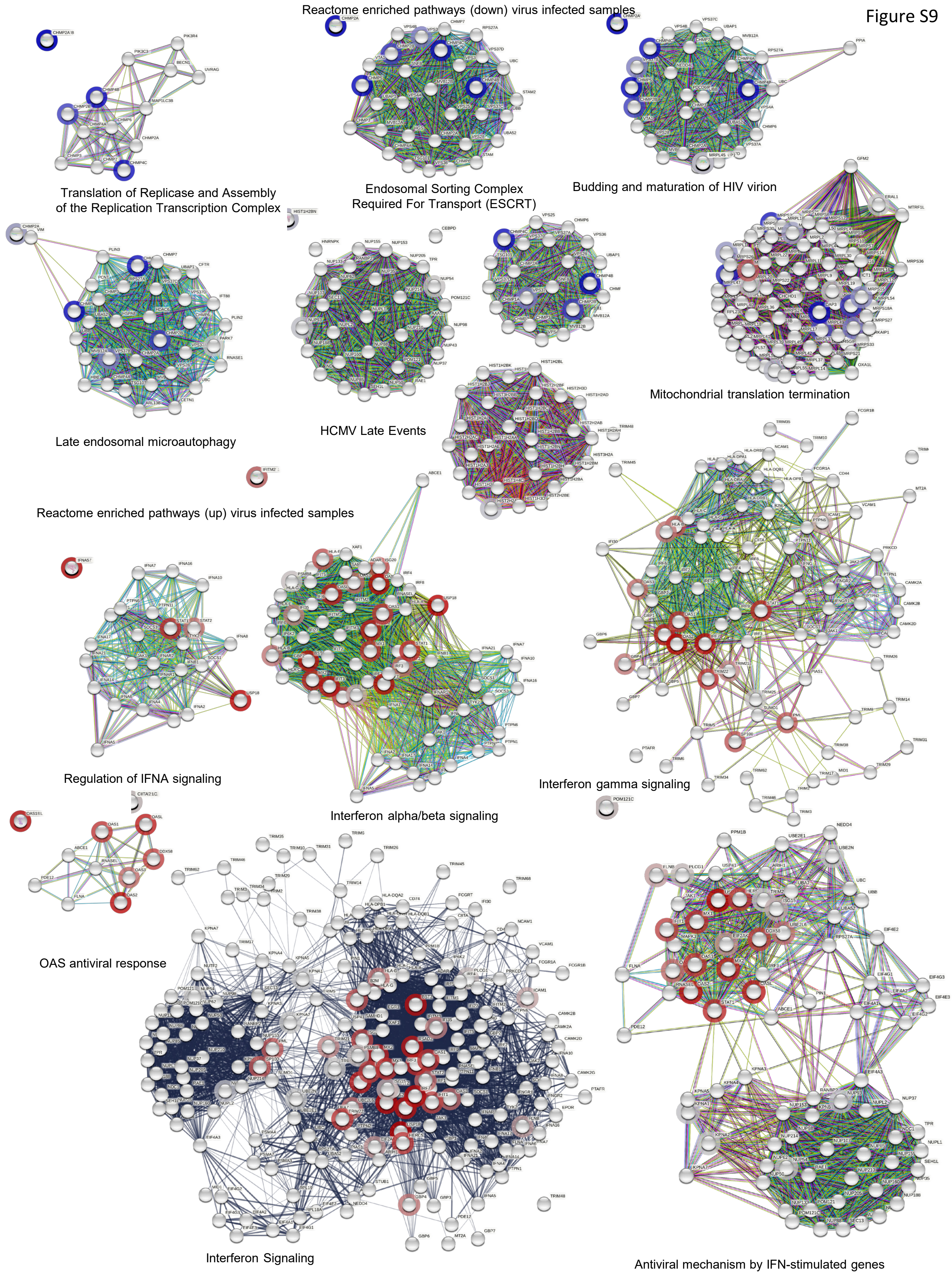
Reactome enriched pathways (down) virus infected samples
Translation of Replicase and Assembly
of the Replication Transcription Complex
Endosomal Sorting Complex
Required For Transport (ESCRT)
Budding and maturation of HIV virion
Late endosomal microautophagy
HCMV Late Events
Mitochondrial translation termination
Reactome enriched pathways (up) virus infected samples
Regulation of IFNA signaling
Interferon alpha/beta signaling
Interferon gamma signaling
OAS antiviral response
Interferon Signaling
Antiviral mechanism by IFN-stimulated genes

Alpha variant		Beta variant		Delta variant		Omicron variant	
Defining mutations		Defining mutations		Defining mutations		Defining mutations	
Nonsynonymous	Synonymous	Nonsynonymous	Synonymous	Nonsynonymous	Synonymous	Nonsynonymous	Synonymous
S:H69-	C241T	S:D80A	G174T	S:T19R	G210T	S:A67V	C241T
S:V70-	C913T	S:D215G	C241T	S:E156-	C241T	S:H69-	C3037T
S:Y144-	C3037T	S:L241-	C3037T	S:F157-	C3037T	S:V70-	T5386G
S:N501Y	C5986T	S:L242-	C28253T	S:R158G	A28271-	S:T95I	T13195C
S:A570D	C14676T	S:A243-		S:L452R	G29742T	S:G142-	C15240T
S:D614G	C15279T	S:K417N		S:T478K		S:V143-	C25000T
S:P681H	T16176C	S:E484K		S:D614G		S:Y144-	C25584T
S:T716I		S:N501Y		S:P681R		S:Y145D	A27259C
S:S982A		S:D614G		S:D950N		S:N211-	C27807T
S:D1118H		S:A701V		ORF1b:P314L		S:L212I	A28271T
ORF1a:T1001I		ORF3a:Q57H		ORF1b:G662S		S:G339D	
ORF1a:A1708D		ORF1a:T265I		ORF1b:P1000L		S:S371L	
ORF1a:I2230T		ORF1a:K1655N		M:I82T		S:S373P	
ORF1a:S3675-		ORF1a:K3353R		N:D63G		S:S375F	
ORF1a:G3676-		ORF1a:S3675-		N:R203M		S:K417N	
ORF1a:F3677-		ORF1a:G3676-		N:D377Y		S:N440K	
N:D3L		ORF1a:F3677-		ORF3a:S26L		S:G446S	
N:R203K		N:T205I		ORF7a:V82A		S:S477N	
N:G204R		ORF1b:P314L		ORF7a:T120I		S:T478K	
N:S235F		E:P71L		ORF8:D119-		S:E484A	
ORF1b:P314L				ORF8:F120-		S:Q493R	
ORF8:Q27*				ORF9b:T60A		S:G496S	
ORF8:R52I						S:Q498R	
ORF8:Y73C						S:N501Y	
						S:Y505H	
						S:T547K	
						S:D614G	
						S:H655Y	
						S:N679K	
						S:P681H	
						S:N764K	
						S:D796Y	
						S:N856K	
						S:Q954H	
						S:N969K	
						S:L981F	
						N:P13L	
						N:E31-	
						N:R32-	
						N:S33-	
						N:R203K	
						N:G204R	
						ORF1a:K856R	
						ORF1a:S2083-	
						ORF1a:L2084I	
						ORF1a:A2710T	
						ORF1a:T3255I	
						ORF1a:P3395H	
						ORF1a:L3674-	
						ORF1a:S3675-	
						ORF1a:G3676-	
						ORF1a:I3758V	
						ORF1b:P314L	
						ORF1b:I1566V	
						ORF9b:P10S	
						ORF9b:E27-	
						ORF9b:N28-	
						ORF9b:A29-	
						E:T9I	
						M:D3G	
						M:Q19E	
						M:A63T	
